# Revegetation by sowing reduces soil bacterial and fungal diversity

**DOI:** 10.1002/ece3.5906

**Published:** 2019-12-08

**Authors:** Chao Wang, Weiwei Zhang, Chunqiao Zhao, Ruishuang Shi, Ruibin Xue, Xiaona Li

**Affiliations:** ^1^ Beijing Research & Development Center for Grasses and Environment Beijing Academy of Agriculture and Forestry Sciences (BAAFS) Beijing China

**Keywords:** bacteria, biodiversity, ecological restoration, fungi, high‐throughput sequencing, revegetation

## Abstract

**Aim:**

The aim of this study was to understand the effects of revegetation on the diversity of bacteria and fungi in soil by sowing a single species and exploring the underlying mechanism.

**Location:**

Beijing, China.

**Taxon:**

Plants and Microbes.

**Methods:**

In a short‐term ecological restoration experiment, one natural recovery treatment and three seed sowing treatments were chosen to assess their effects on the alteration of fungal and bacterial diversity. Plant species richness, abundance, and height were investigated. The diversity of fungi and bacteria was analyzed by high‐throughput sequencing technologies. Linear mixed‐effects model analysis was used to examine the effects of different restoration methods on biodiversity and ecosystem functions. Pearson's correlation analysis, analysis of covariance, and structural equation modeling (SEM) were used to examine the relationship between biodiversity and environmental factors.

**Results:**

Species richness and the Shannon–Wiener Index (*H*′) of plants in the sown treatments were lower than in the natural recovery treatment, especially with sowing of *Medicago sativa* L. Similarly, the sum of the observed species and *H*′ of fungi and bacteria significantly decreased in the sown treatments. Moreover, plant density, community coverage, and soil moisture increased markedly, while soil bulk density decreased in the sown treatments. Importantly, SEM showed that sown treatments reduced the diversity of plants through increasing plant density, while it decreased the diversity of fungi and bacteria through decreasing the plant diversity and increasing soil moisture.

**Main conclusions:**

Our findings confirm that ecological restoration by sowing could improve soil conditions, but may be unfavorable to the amelioration of soil microbial diversity in the short‐term. Restoration practitioners should consider long‐term studies on the dynamics of biodiversity in the above‐ and belowground after revegetation by native species to achieve goals related to biodiversity conservation.

## Graphical abstract

Our study has clearly addressed how and by what mechanism different ecological restoration methods influence the diversity of fungi and bacteria in the soil. We found that restoration by revegetating reduced the diversity of plants through increasing plant density, and decreased the diversity of fungi and bacteria through decreasing the plant diversity and increasing soil moisture.

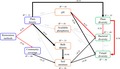

## 1 INTRODUCTION

During the past century, most ecosystems have experienced large‐scale degradation as a result of the increasing influence of human activities, such as agricultural intensification and urbanization (Bullock, Aronson, Newton, Pywell, & Rey Benayas, 2011; Steffen et al., 2015). Ecosystem degradation often leads to a loss of biodiversity (Borer et al., 2014; Gossner et al., 2016; Tscharntke et al., 2012) and a reduction in the variety of ecosystem functions and services available worldwide (Millennium Ecosystem Assessment, 2005), thus causing a growing need for ecological restoration (Jackson & Hobbs, 2009). Ecological restoration efforts often aim to recover an ecosystem by revegetation (Sala et al., 2000). Common methods that are used in revegetation include prohibiting some or all human activities, sowing native species, and transplanting of native seedlings in the field sites (Corlett, 2016; Godefroid et al., 2011). Ecological restoration efforts have diverse effects on biodiversity because of the differences in restoration methods, ecosystem types, climate, and the degree of degradation that has occurred in the past (Barral, Rey Benayas, Meli, & Maceira, 2015; Falk, Schmidt, & Lena, 2014; Martin, 2017; Valliere, Zhang, Sharifi, & Rundel, 2019). For example, the increase of biodiversity in restored areas has been significantly lower than in areas experiencing natural recovery at a global scale (Rey Benayas, Newton, Diaz, & Bullock, 2009). Biodiversity increased more significantly in tropical terrestrial ecosystems than in temperate terrestrial ecosystems after ecological restoration (Rey Benayas et al., 2009). However, most of previous studies have focused on the effects of ecological restoration on biodiversity (Alexander, Aronson, Whaley, & Lamb, 2016; Cao, Shang, Yue, & Ma, 2017; Lu et al., 2018), but less attention has been paid to the effects of different restoration methods, such as natural recovery, sowing plants, and transplanting seedlings (Valliere et al., 2019). Hence, having a comprehensive understanding on the effects of different restoration methods on biodiversity is vital for choosing suitable restoration methods.

Global policy commitments such as the Convention on Biological Diversity (CBD) support restoration actions that are increasingly being implemented throughout the world (CBD, 2012); one of the major goals of ecological restoration is to increase the biodiversity of degraded areas (Jordan, Peters, & Allen, 1988). The effects of restoration on biodiversity in aboveground habitats have been well demonstrated (Felton, Knight, Wood, Zammit, & Lindenmayer, 2010; Ilstedt, Malmer, Verbeeten, & Murdiyarso, 2007). Meta‐analyses in specific ecosystem types, such as forests (Felton et al., 2010) and wetlands (Meli, Rey Benayas, Balvanera, & Martinez, 2014), have reported that biodiversity is altered significantly after ecological restoration. Meta‐analyses at the global scale have found that the biodiversity in restored ecosystems was an average of 44% higher than in degraded ecosystems, but was an average of 14% lower than in ecosystems that experienced natural recovery (Rey Benayas et al., 2009). The diversity of aboveground and belowground ecosystems is well known to be closely correlated with each other (Jing et al., 2015). However, little attention has been paid to the effects of ecological restoration on biodiversity in the belowground. Therefore, investigating the effects of restoration on diversity in the belowground facilitates gaining a comprehensive understanding on the effects of ecological restoration on biodiversity in general.

Soils host the vast majority of life on Earth including microorganisms and animals, such as viruses, bacteria, fungi, protists, nematodes, and earthworms (Geisen et al., 2019). Microorganisms (bacteria and fungi) may serve as possible bio‐indicators for monitoring soil ecosystem functions in close association with changes in the physicochemical and biological conditions during the ecological restoration of degraded areas (Mendez, García, Maestre, & Escudero, 2008; Wang et al., 2012). Previous studies showed that the soil microbial community changed markedly after ecological restoration (Li et al., 2016; Yan et al., 2018). The results from Li et al. (2016) have shown that significant differences existed in the relative abundance of bacteria in some phyla after restoration. Yan et al. (2018) observed that a dramatic shift in the fungal community toward that of the natural fungal community occurred after only 10 years of active native plant revegetation. However, it remains unclear how the diversity of bacteria and fungi changes after revegetation.

The North China Plain covers an area of 440,000 km^2^, with plains accounting for 70% and mountains about 30% of the entire area in this vital agricultural region in China (Song, Deng, Yuan, Wang, & Li, 2015). The second largest area of plains in China, the North China Plain, has experienced rapid ecosystem degradation (Ju, Kou, Zhang, & Christie, 2006; Song et al., 2015). Here, we report on a 1‐year field experiment investigating the influence of different restoration methods on soil microbial community diversity in a degraded farmland area on the North China Plain, based on two restoration methods. The first method involved a natural recovery treatment (stopping the interference from human activities). The second included three sowing treatments, specifically the sowing of *Medicago sativa* L, *Bromus inermis Leyss*, and *Agropyron cristatum* Gaertn. We aimed to explore whether soil microbial diversity in the sown area is significantly higher than that in the natural recovery area, whether the dynamics of microbial diversity are affected by plant diversity in the aboveground, and whether any of the sown treatments can provide a better ecological restoration methods at the local scale.

## 2 METHODS AND MATERIAL

### 2.1 Site description and soil sampling

The experiment started in September, 2017, at Yanqing District, Beijing (116°0′30″E–1′30″E, 40°26′30″N–27′30″N, 492 m above sea level), which is located on the Northern China Plain. The average annual temperature and precipitation from 2000 to 2018 are 8.4°C and 467 mm, respectively. After Beijing became the successful bidder for the 2022 Winter Olympic Games, numerous ecological restoration projects were implemented by the Yanqing Municipal Government while adhering to the concept of hosting a Green Olympics, especially for areas of abandoned farmland. Our study area was in the vicinity of an abandoned canal, covered an area of 27 hm^2^, and was once farmland; however, this land had been abandoned more than 10 years ago because the river ran dry, and construction debris had been abandoned here in some areas. Our experiment was conducted after the construction debris was removed in 2017 (Li et al., 2019). The soil is currently a cinnamon soil (Chinese Soil Taxonomy Research Group, 1995). The dominant species were *Setaria viridis* (L.) Beauv, *Echinochloa crusgalli* (L.) Beauv, and *Artemisia annua* L.

A two restoration methods experiment with a randomized design was conducted in 2017. Twenty seven plots were established in our study, with an area of 100–300 m^2^ per plot; these involved two different restoration methods, including natural recovery treatments (NR, six plots) and sown treatments. The sown treatments including three different species: sown with *Medicago sativa* (MS, nine plots), *Bromus inermis Leyss* (BS, six plots), and *Agropyron cristatum* (AS, six plots; Li et al., 2019). The seeds were selected based on the adaptation of these plants to the local environment and because seeds of these plants are commercially available; seeds were sown without plowing in September, 2017, with the density of 200 seeds per m^2^ for each species. In each plot, plant community was investigated in three randomly selected 1 m × 1 m quadrats in 2018. Three soil cores (diameter 7 cm) were taken in each quadrat and then mixed together into one sample. The soils were kept in a cooler and shipped while refrigerated to the laboratory as quickly as possible, where the soil was sieved to remove roots and stones, then each sample was divided into two parts: one part was for biogeochemical analysis and was stored at 4°C; the second part was immediately packed in polyethylene bags and then immediately submerged in liquid nitrogen for storage in the lab at −80°C prior to DNA extraction (Li et al., 2019).

### 2.2 Vegetation and soil properties analysis

Plant species richness, height, abundance, and coverage were investigated in three 1 × 1 m quadrats for each plot. Soil bulk density (BD) and maximum moisture capacity (MMC) were measured using the soil core; soil pH was measured by a potentiometer after shaking a soil water suspension (1:2.5 water/soil) for 30 min; soil moisture (SM) was measured gravimetrically. Soil available phosphorus (AP) was measured by the sodium bicarbonate leaching‐molybdenum‐antimony colorimetric method (Stahlberg, 1980), total phosphorus (TP) was measured by the sodium hydroxide melting‐molybdenum‐barium colorimetric method (Bowman, 1988), soil organic carbon (SOC) and total nitrogen (TN; Walkley & Black, 1934) were measured by potassium dichromate oxidation and the Kjeldahl method, respectively. Soil available nitrogen (AN) was determined colorimetrically by automated segmented flow analysis. Soil cation exchange capacity and electronic conductivity were measured by the ammonium acetate exchange and conductance methods, respectively. Total and available potassium were measured by ammonium acetate extraction‐atomic absorption spectrophotometry (Li et al., 2019). Biogeochemical data are shown in Table 1.

**Table 1:** Measured soil properties at the depth of 0–10 cm in different restoration methods

Soil property	NR	MS	BS	AS
pH	8.32 ± 0.07b	8.33 ± 0.04b	8.18 ± 0.05a	8.37 ± 0.05b
EC (us/cm)	123.5 ± 13.7	115.5 ± 7.6	134.7 ± 14.3	104.3 ± 9.5
CEC (cmol/kg)	8.39 ± 0.80	8.69 ± 0.93	9.70 ± 0.45	7.86 ± 0.96
SOC (g/kg)	11.40 ± 1.74b	7.75 ± 1.61ab	9.73 ± 0.78ab	6.10 ± 0.80a
AP (mg/kg)	10.48 ± 2.49	10.96 ± 2.20	9.52 ± 0.91	11.12 ± 3.25
AK (mg/kg)	146.2 ± 14.3	149.1 ± 14.6	171.5 ± 9.8	128.4 ± 21.7
AN (mg/kg)	36.55 ± 6.18b	24.96 ± 3.09a	28.49 ± 2.75ab	19.60 ± 2.27a
TN (%)	0.64 ± 0.07b	0.41 ± 0.06a	0.54 ± 0.04ab	0.37 ± 0.04a
TP (%)	0.43 ± 0.03	0.51 ± 0.05	0.55 ± 0.03	0.51 ± 0.04
SM (%)	7.48 ± 0.33a	8.70 ± 1.10b	8.11 ± 1.08ab	8.22 ± 1.31ab
MMC (%)	25.89 ± 1.34a	27.05 ± 1.36ab	33.82 ± 1.89b	28.76 ± 3.69ab
TSP (cm^3^/cm^3^)	0.57 ± 0.08	0.53 ± 0.05	0.48 ± 0.07	0.50 ± 0.10
BD (g/cm^3^)	1.41 ± 0.03b	1.37 ± 0.03ab	1.24 ± 0.05a	1.32 ± 0.05a

Note

Different letters (a, b, and c) within the same row indicate significant differences among restoration methods. Data shown are means ± *SE* (*n* = 6 or 9).

Abbreviations: AK, available potassium; AN, available nitrogen; AP, available phosphorus; AS, *Agropyron cristatum* Gaertn sowing; BD, bulk density; BS, *Bromus inermis Leyss* sowing; CEC, cation exchange capacity; EC, electrical conductivity; MMC, maximum moisture capacity; MS, *Medicago sativa* L sowing; NR, natural recovery; SM, soil moisture; SOC, soil organic carbon; TN, total nitrogen; TP, total phosphorus; TSP, total soil porosity.

### 2.3 DNA extraction and PCR amplification

Microbial DNA was extracted from samples using the FastDNA^®^SPIN Kit for soil. The final DNA concentration and purification were determined by NanoDrop 2000 UV‐vis spectrophotometer (Thermo Scientific), and DNA quality was checked by 1% agarose gel electrophoresis. The V3‐V4 hypervariable regions of the bacteria 16S rRNA gene were amplified with primers 338F (5′‐ACTCCTACGGGAGGCAGCAG‐3′) and 806R (5′‐GGACTACHVGGGTWTCTAAT‐3′) and the fungi 18S rRNA gene was amplified with primers ITS1F (5′‐CTTGGTCATTTAGAGGAAGTAA‐3′) and ITS2R (5′‐GCTGCGTTCTTCATCGATGC‐3′) by thermocycler PCR system. PCRs were conducted by the following program: 3 min of denaturation at 95°C, 37 cycles of 30 s at 95°C, 30 s for annealing at 55°C, 45 s for elongation at 72°C, and then a final extension at 72°C for 10 min. The PCRs were performed in triplicate 20 μl mixture containing 4 μl of 5 × FastPfu Buffer, 2 μl of 2.5 mM dNTPs, 0.8 μl of each primer (5 μM), 0.4 μl of FastPfu Polymerase, and 10 ng of template DNA. The resulted PCR products were extracted from a 2% agarose gel and further purified by the AxyPrep DNA Gel Extraction Kit (Axygen Biosciences) and quantified using QuantiFluor™‐ST (Promega) according to the manufacturer's protocol.

Purified amplicons were pooled in equimolar mixtures and paired‐end sequenced (2 × 300) on an Illumina MiSeq platform (Illumina) according to the standard protocols by Majorbio Bio‐Pharm Technology Co. Ltd.

### 2.4 Processing of sequencing data

Sequences were quality‐filtered by Trimmomatic and merged by FLASH with the following criteria: (a) the reads were truncated at any site receiving an average quality score <20 over a 50 bp sliding window; (b) sequences whose overlap was longer than 10 bp were merged according to their overlap with mismatch of no more than 2 bp; (c) sequences of each sample were separated according to barcodes and primers, and reads containing ambiguous bases were removed. Operational taxonomic units (OTUs) were clustered with 97% similarity cutoff using UPARSE (version 7.1 http://drive5.com/uparse/) with a novel “greedy” algorithm that performs chimera filtering and OTU clustering simultaneously. We rarified the abundance matrix to 1,000 sequences per sample to obtain normalized relative abundances.

### 2.5 Statistical analyses

The Shannon–Wiener Index (*H*′) was used to quantify the diversity of plants and microbes, using $H^{\prime }=-\sum_{i=1}^{S}\frac{n_{i}}{N}\left(\ln\frac{n_{i}}{N}\right)$, where *S* is the sum of species (for microbes, Sobs represents the sum of observed species), *n_i_* is the number of *i*th species, and *N* is the numbers of individuals. The Pielou Evenness Index was used to assess the evenness of plant density, $J=\frac{H^{\prime }}{\ln\left(S\right)}$. Dominant species included those with relative abundances higher than 5% (Ma et al., 2017). The effects of restoration methods on biodiversity were examined using linear mixed‐effects models (R‐function “lme”), in which restoration methods are used as the fixed effects, and the plot is used as the random effects. The relationship between the biotic and abiotic factors was investigated by Pearson's correlation and analysis of covariance. Structural equation modeling (SEM) was employed to evaluate the hypothesized underlying factors that influence biodiversity in above‐ and belowground under different restoration methods (Wang et al., 2017; Wang & Tang, 2019) using the package “piecewise‐SEM” in R (Shipley, 2000). The restoration methods were converted to numeric variables in the SEM, where 0 represents natural recovery, and 1 represent sown treatments. The model was assessed by Fisher's *C* statistic, Akaike information criterion (AIC) and AICc values, and *p*‐values.

All statistical analyses and graphs were prepared in R 3.2.2 (R Core Team, 2018). Differences were considered to be statistically significant at *p* ≤ .05.

## 3 RESULTS

### 3.1 The effects of restoration methods on species diversity

**Figure 1:**
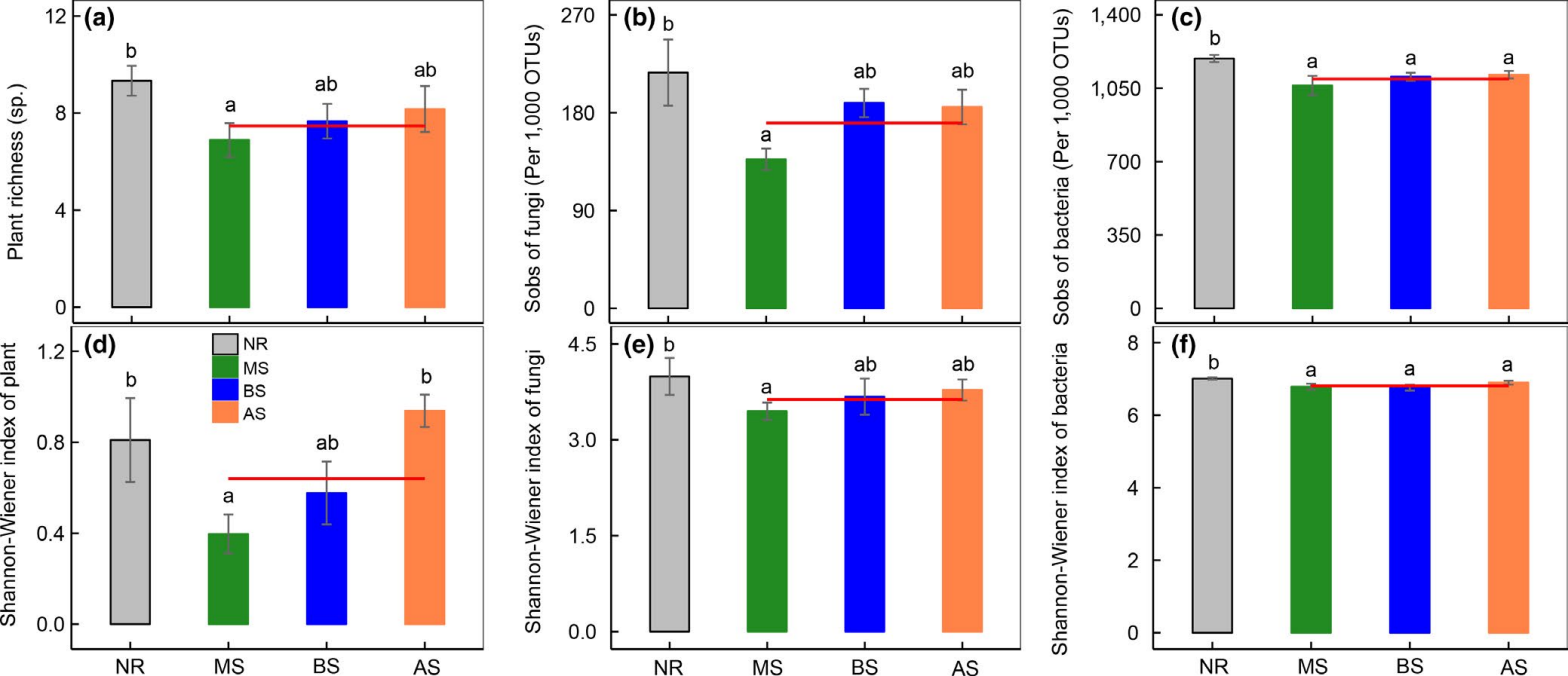
Diversity of plant, fungi, and bacteria in different restoration methods. Shown are (a) plant richness; (b) Sobs of fungi; (c) Sobs of bacteria; (d) Shannon–Wiener Index of plant; (e) Shannon–Wiener index of fungi; (f) Shannon–Wiener index of bacteria. The red line represents the average of three sown treatments. Different lowercase letters above the standard error bars indicate significant differences among treatments (*p* < .05). AS, *Agropyron cristatum* Gaertn sowing; BS, *Bromus inermis Leyss* sowing; MS, *Medicago sativa* L sowing; NR, natural recovery; Sobs, sum of observed species. Vertical bars represent the *SEM* (*n* = 6 or 9)

**Figure 2:**
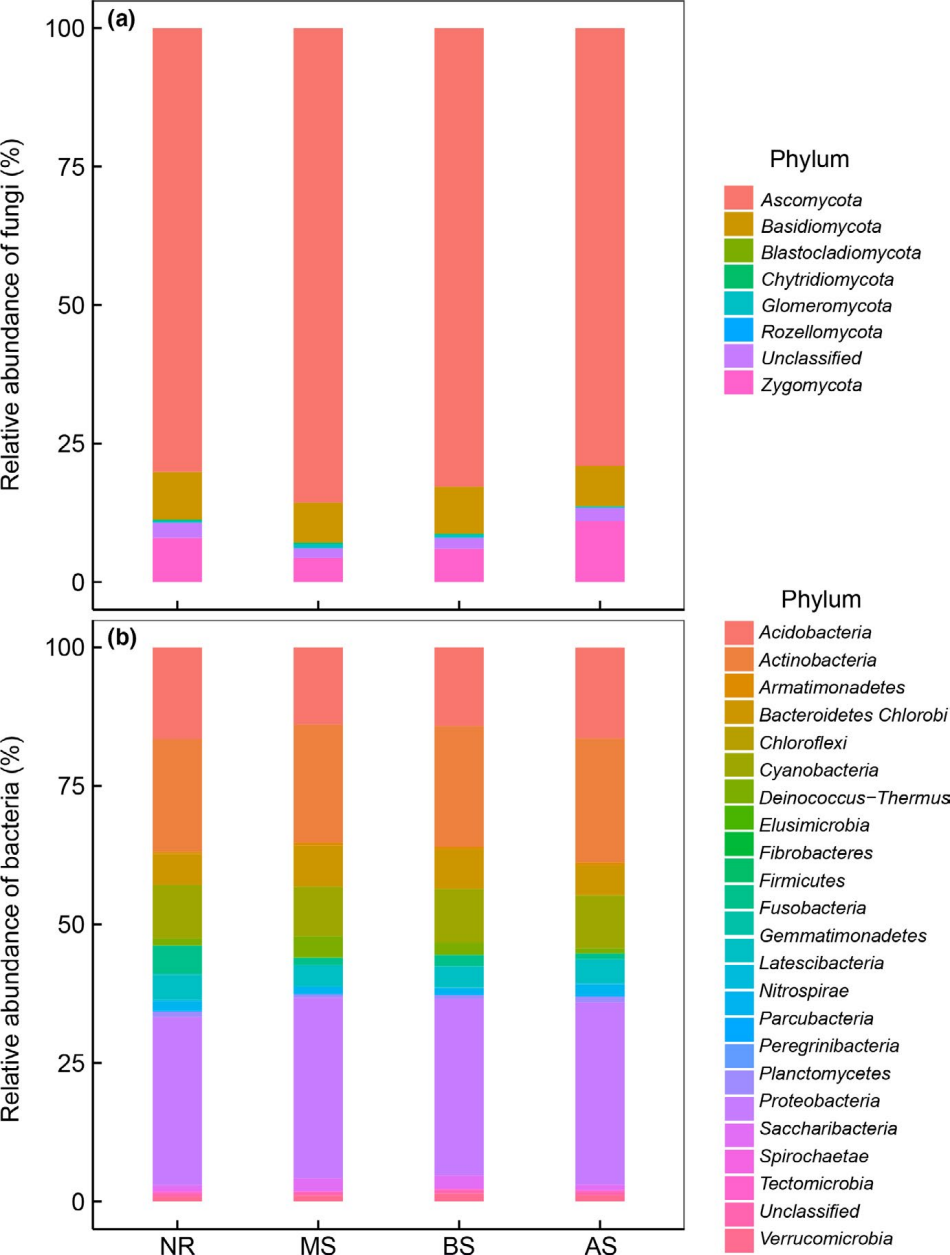
Relative abundance of fungi and bacteria at phylum levels in different restoration methods. Shown are relative abundance of (a) fungi and (b) bacteria. AS, *Agropyron cristatum* Gaertn sowing; BS, *Bromus inermis Leyss* sowing; MS, *Medicago sativa* L sowing; NR, natural recovery

**Figure 3:**
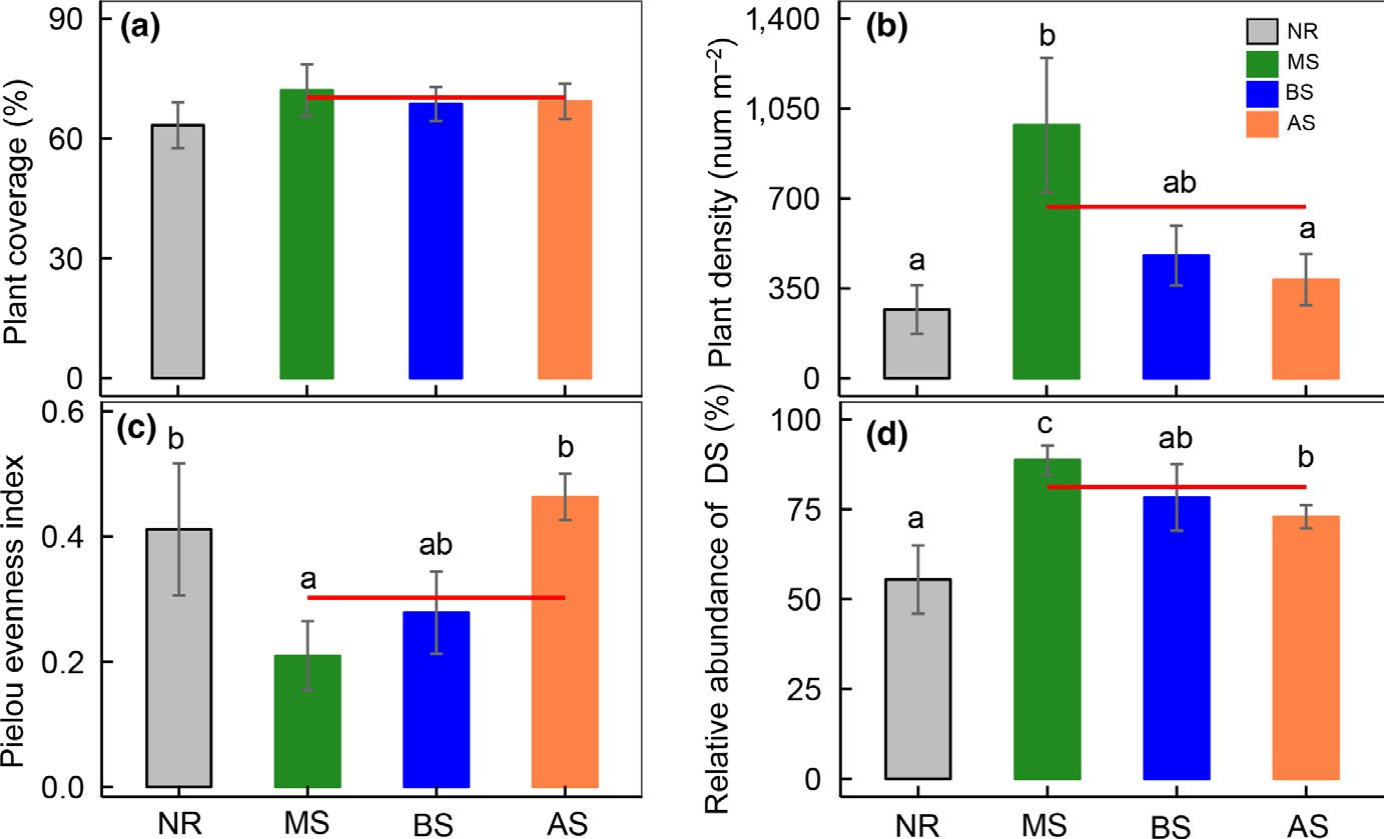
Community structure of plant in different restoration methods. Shown are (a) plant coverage; (b) plant density; (c) Pielou Evenness of plant; (d) relative abundance of dominant species. The red line represents the average of three sown treatments. Different lowercase letters above the standard error bars indicate significant differences among treatments (*p* < .05). AS, *Agropyron cristatum* Gaertn sowing; BS, *Bromus inermis Leyss* sowing; DS, dominant species; MS, *Medicago sativa* L sowing; NR, natural recovery. Vertical bars represent the *SEM* (*n* = 6 or 9)

Over the 1‐year experimental period, the using of sowing for ecological restoration significantly influenced the biodiversity in soil, but the effects varied in the different sown treatments (Figure 1 and Table 1). Species richness (Sobs of fungi) and the Shannon–Wiener Index (*H*′) of plants and fungi after sowing *Medicago sativa* (MS) were significantly lower than in the natural recovery (NR) treatment; specifically, plant species richness decreased 26.2% (three species [spp.]) and the Sobs of fungi decreased 36.8% (80 spp.) in the MS treatments. Meanwhile, no significant difference in the diversity of plants and fungi was observed between NR and *Bromus inermis Leyss* (BS) and *Agropyron cristatum* (AS) sowing treatments (Figure 1). When compared with the NR treatment, the Sobs and *H*′ of bacteria decreased 10.8% (133 spp.) and 3.2% in the sown treatments, respectively (Figure 1c,f, Table S1). In addition, our results show that the relative abundances of fungi and bacteria at the phylum level were different among the two restoration methods (Figure 2). For the fungi, the relative abundance of *Ascomycota* in the MS treatment was significantly larger than in the NR treatment, while that of *Zygomycota* was smaller in the MS treatment (Figure 2a and Table S2). For the bacteria, the relative abundance of *Acidobacteria* in the MS and BS treatment was significantly lower than that in NR. When compared with NR treatment, the relative abundance of *Firmicutes* significantly decreased, while that of the *Proteobacteria* increased after sowing (Figure 2b and Table S3). Moreover, we found that the coverage of the plant community in different restoration methods showed no significant difference (Figure 3a), while the plant density and relative abundance of dominant species increased by 149% and 46.3% after sowing (Figure 3b,d).

Soil properties changed significantly after sowing (Table 1). Soil organic carbon, total nitrogen, available nitrogen, and bulk density at the top soil decreased significantly, while soil moisture and maximum moisture capacity increased significantly in the sown treatments (Table 1). However, soil pH, electrical conductivity, cation exchange capacity, available phosphorus, and total phosphorus showed no significant differences between the NR and sown treatments (Table 1).

### 3.2 Ecological factors influencing diversity of bacteria and fungi

**Figure 4:**
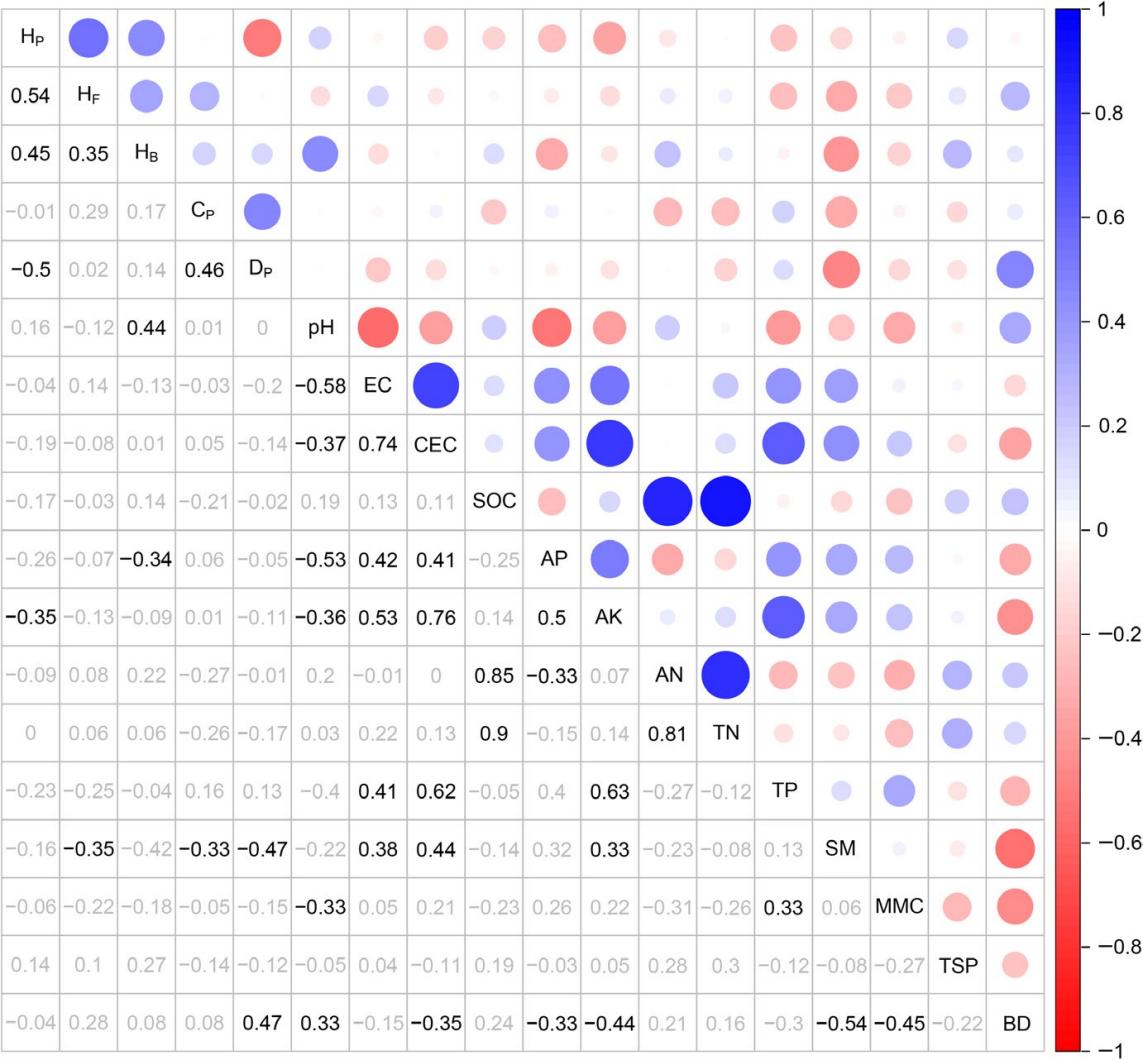
Relationship between the Shannon–Wiener Index of species and environmental factors. The size of each circle represents the correlation coefficient of two parameters, blue and red circles represent positive and negative relationships, respectfully. The numbers below the triangles are the coefficients where black and gray numbers represent significant and insignificant relationships, respectfully. AK, available potassium; AN, available nitrogen; AP, available phosphorus; BD, bulk density; CEC, cation exchange capacity; *C*_p_, plant coverage; *D*_p_, plant density; EC, electrical conductivity; *H*_b_, Shannon–Wiener Index of bacteria; *H*_f_, Shannon–Wiener Index of fungi; *H*_p_, Shannon–Wiener Index of plant; MMC, maximum moisture capacity; SM, soil moisture; SOC, soil organic carbon; TN, total nitrogen; TP, total phosphorus; TSP, total soil porosity

**Figure 5:**
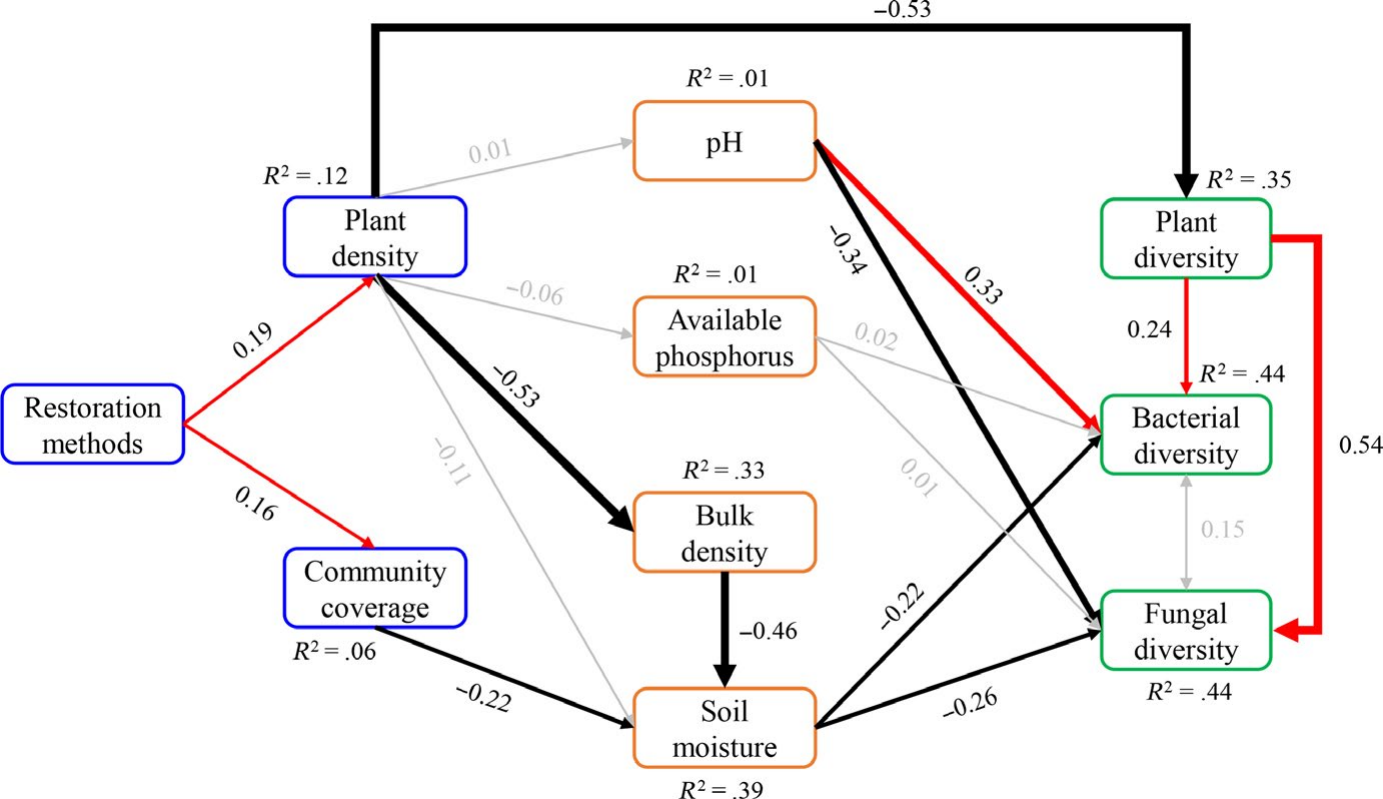
A structural equation modeling of restoration methods impact on biodiversity. The structural equation modeling considered all significant pathways through which restoration on influence biodiversity. Red and black arrows represent significant positive and negative pathways, respectively, and gray arrows indicate non‐significant pathways. Numbers indicate the standard path coefficients. Arrow width is proportional to the strength of the relationship. *R* ^2^ represents the proportion of variance explained for each dependent variable. Fisher.c = 150.5, *p* = .06, Akaike information criterion (AIC) = 224.5, AICc = 400.25, *n* = 27

Pearson's correlation revealed that diversity of plants was positively correlated with the diversity of fungi and bacteria, while it was negatively correlated with plant density and available potassium (Figure 4 and Table S4). The diversity of fungi was positively correlated with the diversity of bacteria, but negatively correlated with soil moisture (Figure 4 and Table S4). A significant positive relationship was found between bacterial diversity and soil pH, while a significant negative relationship was found between bacterial diversity and available phosphorus (Figure 4 and Table S4). Structural equation modeling showed that plant density explained 35% of the variation in plant diversity. The combination of soil pH and soil moisture, and the plant diversity jointly explained 44% and 44% of the variation in the diversity of fungi and bacteria, respectively (Figure 5). The negative effects of sown treatments on plant diversity were mainly through its positive effects on plant density, while the negative effects of sown treatments on the diversity of fungi and bacteria were mainly through its negative effects on plant diversity and positive effect on soil moisture (Figure 5).

## 4 DISCUSSION

Ecological restoration actions are increasingly being implemented throughout the world (Jackson, Lopoukhine, & Hillyard, 1995; Jackson & Hobbs, 2009). Ecologists have been exploring the re‐establishment of the biodiversity and ecosystem function in the degraded ecosystem through revegetation (Martin, 2017; Rey Benayas et al., 2009). Three aspects of our study, however, distinguish it from previous studies on the effects of ecological restoration on biodiversity. First, our study was conducted to examine the effects of different restoration methods on biodiversity. Second, we examined the effects of ecological restoration on the diversity of plants, fungi, and bacteria. Third and most important, our study demonstrated that using sowing seeds for ecological restoration could improve the soil conditions but significantly reduced the diversity of fungi and bacteria.

### 4.1 The effects of sowing on community structures and soil properties

Many studies, including those based on field observations (Alexander et al., 2016; Cao et al., 2017; Sheng, Zhen, Xiao, & Hu, 2019) and meta‐analyses (Rey Benayas et al., 2009), have shown that ecological restoration tends to improve the functioning of ecosystem. In our study, we found community traits and soil properties improved after sowing, such as an increase in community coverage, plant density, soil moisture, and reduction in soil bulk density (Table 1 and Figure 3). Given that plant density increased significantly in the *Medicago sativa* sowing treatment, as also found in other studies (Sun et al., 2015), one might hypothesize that the increase in plant density was the result of a larger seed bank following sowing. However, the experimental site is located at northern China, where a limited amount of soil nitrogen is available (Gruber & Galloway, 2008; Wang et al., 2014). Legumes can fix nitrogen by means of symbionts (Bordeleau & Prévost, 1994), making the fixed nitrogen available for other plants and organisms growth under N‐limited conditions (Temperton, Mwangi, Scherer‐Lorenzen, Schmid, & Buchmann, 2007). Hence, we believe the ability of *Medicago sativa* to fix nitrogen caused a significant increase in plant density after sowing, while this did not occur in the *Bromus inermis Leyss* and *Agropyron cristatum* sowing treatments.

Soil moisture and bulk density are the basic features of soil and could be influenced by the dynamics of plant density and root biomass (Liu et al., 2018). The findings in our study showed that sowing resulted in reduced bulk density and increased soil moisture, which resulted in an increase in plant density (Figure 5). Where compared with the natural recovery treatment, plant density increased significantly after sowing, which may have led to an increase in root biomass (Chapin, 1980); the increase in root biomass may have caused a decrease in bulk density and an increase in soil moisture (Craig & Fraterrigo, 2017; Guzman & Al‐Kaisi, 2011). Moreover, with the increase in plant density, the decomposition rate of soil carbon may increase significantly due to the enhancement of rhizosphere priming effects (Zhu et al., 2014), and this may then lead to a decline in soil organic carbon after sowing.

### 4.2 Diversity of fungi and bacteria decreases after sowing

A growing body of evidence suggests that the plant diversity in a sowing or planting area tends to be lower than in an area that recovers naturally from disturbance (Felton et al., 2010). Our findings reinforce this idea. An important characteristic of sowing was that the relative abundance of dominant species was significantly greater than that in the area of natural recovery (Figure 3), a finding that has also been reported in several other studies (Pywell et al., 2003; Rey Benayas et al., 2009). In degraded ecosystems, the sown species had a greater ability to acquire nutrients and light (Wang et al., 2012), and quickly became the dominant species of community (Figure 3d), which left narrower niches available for the colonization of other species. Hence, few species that both happen to be producing propagules and can disperse to the site will colonize, and thus the community will consist of only those few species capable of quickly reaching maturity (Connell, 1978).

Sowing not only had a negative effect on plant diversity (Cao et al., 2017; Rey Benayas et al., 2009), but also decreased the diversity of fungi and bacteria. Our findings revealed that the diversity of fungi and bacteria decreased significantly in the sown treatments for two reasons. First, a close relationship exists between plants and soil microbes, which is based on plant litter and root exudates (Jing et al., 2015; Mcdaniel, Tiemann, & Grandy, 2016). The decrease of plant diversity in the sown treatments was followed by a decline in the diversity of plant litter and root exudates, the reduction in heterogeneity of resources may induce a reduction of microbial diversity (He, Bazzaz, & Schmid, 2002; Prober et al., 2015), and thus led to a positive relationship between plant diversity and the diversity of fungi and bacteria. Secondly, the sown treatments altered the diversity of soil microbes through changing soil properties, such as soil moisture (Fierer & Jackson, 2006). Soil moisture increased with the increase of plant density in the sown treatments, which may have altered the community structure of fungi and bacteria, and then resulted in a significant decrease in the relative abundance and richness of drought‐tolerant species, such as *Zygomycota* and *Firmicutes* (Table S5; Wrighton et al., 2008); thus, the sown treatments may cause a negative relationship to develop between soil moisture and the diversity of fungi and bacteria.

### 4.3 Limitations of the current study

Spatial distribution of construction rubbish may have contributed to between‐plot variability of biodiversity in the above‐ and belowground. The effects of restoration methods on the diversity of fungi and bacteria mainly occurred through changes in plant density, species richness of plants, and soil moisture. However, the large spatial heterogeneity of plant density (42–2,221 stem/m^2^), plant species richness (3–12 species/m^2^), and soil moisture (4.03%–16.16%) may conceal the effects of restoration methods on the diversity of fungi and bacteria; thus more replications are needed to decrease the influence of site heterogeneity in the future. Moreover, the driver of the observed heterogeneity in fungal and bacterial diversity may change along time scales (Gao et al., 2019), especially in farmland, thus long‐term experiments are needed to evaluate the effects of different restoration methods on the diversity of fungi and bacteria in the soil.

## 5 CONCLUSIONS

Because human activities continue to cause the loss of biodiversity and habitats, adaptive management approaches that enabled practitioners to establish stable plant communities are becoming increasingly important. Our results provide further evidence that revegetation by sowing of a single species led to a reduction in the diversity of fungi and bacteria in soil in the short‐term. Importantly, the decrease in the diversity of fungi and bacteria in the soil was determined by a reduction in plant diversity. Further research that explores the application of sowing with a greater diversity of species and studies using multiple native plant species that can coexist will be useful in determining the use of this approach in biodiversity conservation.

## CONFLICT OF INTEREST

None declared.

## AUTHORS CONTRIBUTION

XL and CW conceived and designed the research; XL, WZ, CZ, RS, and RX performed the experiments; CW analyzed the data, wrote and edited the manuscript.

## Data Availability

The data that support the findings of this study are openly available at https://doi.org/10.5061/dryad.gtht76hh3.

## References

[ece35906-bib-0001] Alexander, S. , Aronson, J. , Whaley, O. , & Lamb, D. (2016). The relationship between ecological restoration and the ecosystem services concept. Ecology and Society, 21, 1–9. 10.5751/ES-08288-210134 27668001

[ece35906-bib-0002] Barral, M. P. , Rey Benayas, J. M. , Meli, P. , & Maceira, N. O. (2015). Quantifying the impacts of ecological restoration on biodiversity and ecosystem services in agroecosystems: A global meta‐analysis. Agriculture, Ecosystems & Environment, 202, 223–231. 10.1016/j.agee.2015.01.009

[ece35906-bib-0003] Bordeleau, L. , & Prévost, D. (1994). Nodulation and nitrogen fixation in extreme environments. Plant & Soil, 161, 115–125. 10.1007/BF02183092

[ece35906-bib-0004] Borer, E. T. , Seabloom, E. W. , Gruner, D. S. , Harpole, W. S. , Hillebrand, H. , Lind, E. M. , … Yang, L. H. (2014). Herbivores and nutrients control grassland plant diversity via light limitation. Nature, 508, 517–520. 24670649 10.1038/nature13144

[ece35906-bib-0005] Bowman, R. A. (1988). A rapid method to determine total phosphorus in soils. Soil Science Society of America Journal, 52, 1301–1304. 10.2136/sssaj1988.03615995005200050016x

[ece35906-bib-0006] Bullock, J. M. , Aronson, J. , Newton, A. C. , Pywell, R. F. , & Rey Benayas, J. M. (2011). Restoration of ecosystem services and biodiversity: Conflicts and opportunities. Trends in Ecology & Evolution, 26, 541–549. 10.1016/j.tree.2011.06.011 21782273

[ece35906-bib-0007] Cao, S. , Shang, D. , Yue, H. , & Ma, H. (2017). A win‐win strategy for ecological restoration and biodiversity conservation in Southern China. Environmental Research Letters, 12, 044004 10.1088/1748-9326/aa650c

[ece35906-bib-0008] CBD (2012). Strategic plan for biodiversity 2011–2020 and the Aichi targets. Retrieved from http://www.cbd.int/doc/strategic-plan/2011-2020/Aichi-Targets-EN.pdf

[ece35906-bib-0009] Chapin, F. S. III (1980). The mineral nutrition of wild plants. Annual Review of Ecology, Evolution, and Systematics, 11, 233–260. 10.1146/annurev.es.11.110180.001313

[ece35906-bib-0010] Chinese Soil Taxonomy Research Group (1995). Chinese Soil Taxonomy (pp. 58–147). Beijing, China: Science Press.

[ece35906-bib-0011] Connell, J. H. (1978). Diversity in tropical rain forest and coral reefs. Science, 199, 1302–1310. 17840770 10.1126/science.199.4335.1302

[ece35906-bib-0012] Corlett, R. T. (2016). Restoration, reintroduction, and rewilding in a changing world. Trends in Ecology & Evolution, 31, 453–462. 10.1016/j.tree.2016.02.017 26987771

[ece35906-bib-0013] Craig, M. E. , & Fraterrigo, J. M. (2017). Plant‐microbial competition for nitrogen increases microbial activities and carbon loss in invaded soils. Oecologia, 184, 583–596. 10.1007/s00442-017-3861-0 28421325

[ece35906-bib-0014] Falk, J. M. , Schmidt, N. M. , & Lena, S. (2014). Effects of simulated increased grazing on carbon allocation patterns in a high arctic mire. Biogeochemistry, 119, 229–244. 10.1007/s10533-014-9962-5

[ece35906-bib-0015] Felton, A. , Knight, E. , Wood, J. , Zammit, C. , & Lindenmayer, D. (2010). A meta‐analysis of fauna and flora species richness and abundance in plantations and pasture lands. Biological Conservation, 143, 545–554. 10.1016/j.biocon.2009.11.030

[ece35906-bib-0016] Fierer, N. , & Jackson, R. B. (2006). The diversity and biogeography of soil bacterial communities. Proceedings of the National Academy of Sciences of the United States of America, 103, 626–631. 10.1073/pnas.0507535103 16407148 PMC1334650

[ece35906-bib-0017] Gao, Q. , Yang, Y. , Feng, J. , Tian, R. , Guo, X. , Ning, D. , … Zhou, J. (2019). The spatial scale dependence of diazotrophic and bacterial community assembly in paddy soil. Global Ecology and Biogeography, 28, 1093–1105. 10.1111/geb.12917

[ece35906-bib-0018] Geisen, S. , Briones, M. J. I. , Gan, H. , Behan‐Pelletier, V. M. , Friman, V.‐P. , de Groot, G. A. , … Wall, D. H. (2019). A methodological framework to embrace soil biodiversity. Soil Biology and Biochemistry, 136, 107536 10.1016/j.soilbio.2019.107536

[ece35906-bib-0019] Godefroid, S. , Piazza, C. , Rossi, G. , Buord, S. , Stevens, A.‐D. , Aguraiuja, R. , … Vanderborght, T. (2011). How successful are plant species reintroductions? Biological Conservation, 144, 672–682. 10.1016/j.biocon.2010.10.003

[ece35906-bib-0020] Gossner, M. M. , Lewinsohn, T. M. , Kahl, T. , Grassein, F. , Boch, S. , Prati, D. , … Allan, E. (2016). Land‐use intensification causes multitrophic homogenization of grassland communities. Nature, 540, 266–269. 10.1038/nature20575 27919075

[ece35906-bib-0021] Gruber, N. , & Galloway, J. N. (2008). An Earth‐system perspective of the global nitrogen cycle. Nature, 451, 293–296. 10.1038/nature06592 18202647

[ece35906-bib-0022] Guzman, J. G. , & Al‐Kaisi, M. M. (2011). Landscape position effect on selected soil physical properties of reconstructed prairies in southcentral Iowa. Journal of Soil and Water Conservation, 66, 183–191. 10.2489/jswc.66.3.183

[ece35906-bib-0023] He, J.‐S. , Bazzaz, F. A. , & Schmid, B. (2002). Interactive effects of diversity, nutrients and elevated CO_2_ on experimental plant communities. Oikos, 97, 337–348. 10.1034/j.1600-0706.2002.970304.x

[ece35906-bib-0024] Ilstedt, U. , Malmer, A. , Verbeeten, E. , & Murdiyarso, D. (2007). The effect of afforestation on water infiltration in the tropics: A systematic review and meta‐analysis. Forest Ecology and Management, 251, 45–51. 10.1016/j.foreco.2007.06.014

[ece35906-bib-0025] Jackson, L. L. , Lopoukhine, N. , & Hillyard, D. (1995). Ecological restoration: A definition and comments. Restoration Ecology, 3, 71–75. 10.1111/j.1526-100X.1995.tb00079.x

[ece35906-bib-0026] Jackson, S. T. , & Hobbs, R. J. (2009). Ecological restoration in the light of ecological history. Science, 325, 567–569. 10.1126/science.1172977 19644108

[ece35906-bib-0027] Jing, X. , Sanders, N. J. , Shi, Y. U. , Chu, H. , Classen, A. T. , Zhao, K. E. , … He, J.‐S. (2015). The links between ecosystem multifunctionality and above‐and belowground biodiversity are mediated by climate. Nature Communications, 6, 8159 10.1038/ncomms9159 PMC4569729 26328906

[ece35906-bib-0028] Jordan, W. R. , Peters, R. L. , & Allen, E. B. (1988). Ecological restoration as a strategy for conserving biological diversity. Environmental Management, 12, 55–72. 10.1007/BF01867377

[ece35906-bib-0029] Ju, X. , Kou, C. , Zhang, F. , & Christie, P. (2006). Nitrogen balance and groundwater nitrate contamination: Comparison among three intensive cropping systems on the North China Plain. Environmental Pollution, 143, 117–125. 10.1016/j.envpol.2005.11.005 16364521

[ece35906-bib-0030] Li, X. , Zhang, W. , Zhao, C. , Song, J. , Shi, R. , Xue, R. , & Wang, C. (2019). Plant diversity and soil physicochemical properties in the wasteland of Yanqing District. Acta Agrestia Sinica, 23, 695–701.

[ece35906-bib-0031] Li, Y. , Jia, Z. , Sun, Q. , Zhan, J. , Yang, Y. , & Wang, D. (2016). Ecological restoration alters microbial communities in mine tailings profiles. Scientific Reports, 6, 25193 10.1038/srep25193 27126064 PMC4850430

[ece35906-bib-0032] Liu, H. , Mi, Z. , Lin, L. I. , Wang, Y. , Zhang, Z. , Zhang, F. , … He, J.‐S. (2018). Shifting plant species composition in response to climate change stabilizes grassland primary production. Proceedings of the National Academy of Sciences of the United States of America, 115, 4051–4056. 10.1073/pnas.1700299114 29666319 PMC5910805

[ece35906-bib-0033] Lu, F. , Hu, H. , Sun, W. , Zhu, J. , Liu, G. , Zhou, W. , … Yu, G. (2018). Effects of national ecological restoration projects on carbon sequestration in China. Proceedings of the National Academy of Sciences of the United States of America, 115, 4039–4044. 10.1073/pnas.1700294115 29666317 PMC5910802

[ece35906-bib-0034] Ma, Z. , Liu, H. , Mi, Z. , Zhang, Z. , Wang, Y. , Xu, W. , … He, J. S. (2017). Climate warming reduces the temporal stability of plant community biomass production. Nature Communications, 8, 15378. 10.1038/ncomms15378 PMC5436222 28488673

[ece35906-bib-0035] Martin, D. M. (2017). Ecological restoration should be redefined for the twenty‐first century. Restoration Ecology, 25, 668–673. 10.1111/rec.12554 29400359 PMC5792077

[ece35906-bib-0036] Mcdaniel, M. D. , Tiemann, L. K. , & Grandy, A. S. (2016). Does agricultural crop diversity enhance soil microbial biomass and organic matter dynamics? A meta‐analysis. Ecological Applications, 24, 560–570. 10.1890/13-0616.1 24834741

[ece35906-bib-0037] Meli, P. , Rey Benayas, J. M. , Balvanera, P. , & Martinez, R. M. (2014). Restoration enhances wetland biodiversity and ecosystem service supply, but results are context‐dependent: A meta‐analysis. PLoS ONE, 9, e93507 10.1371/journal.pone.0093507 24743348 PMC3990551

[ece35906-bib-0038] Mendez, M. , García, D. , Maestre, F. T. , & Escudero, A. (2008). More ecology is needed to restore Mediterranean ecosystems: A reply to Valladares and Gianoli. Restoration Ecology, 16(2), 210–216.

[ece35906-bib-0039] Millennium Ecosystem Assessment (2005). Ecosystems and human well‐being: Synthesis. Washington, DC: Island Press.

[ece35906-bib-0040] Prober, S. M. , Leff, J. W. , Bates, S. T. , Borer, E. T. , Firn, J. , Harpole, W. S. , … Fierer, N. (2015). Plant diversity predicts beta but not alpha diversity of soil microbes across grasslands worldwide. Ecology Letters, 18, 85–95. 10.1111/ele.12381 25430889

[ece35906-bib-0041] Pywell, R. F. , Bullock, J. M. , Roy, D. B. , Warman, L. I. Z. , Walker, K. J. , & Rothery, P. (2003). Plant traits as predictors of performance in ecological restoration. Journal of Applied Ecology, 40, 1–10. 10.1046/j.1365-2664.2003.00762.x

[ece35906-bib-0042] R Core Team (2018). R: A language and environment for statistical computing. Vienna, Austria: R Foundation for Statistical Computing.

[ece35906-bib-0043] Rey Benayas, J. M. , Newton, A. C. , Diaz, A. , & Bullock, J. M. (2009). Enhancement of biodiversity and ecosystem services by ecological restoration: A meta‐analysis. Science, 325, 1121–1124. 10.1126/science.1172460 19644076

[ece35906-bib-0044] Sala, O. E. , Chapin, F. S. III , Armesto, J. J. , Berlow, E. , Bloomfield, J. , Dirzo, R. , … Wall, D. H. (2000). Global biodiversity scenarios for the Year 2100. Science, 287, 1–5. 10.1126/science.287.5459.1770 10710299

[ece35906-bib-0045] Sheng, W. , Zhen, L. , Xiao, Y. , & Hu, Y. (2019). Ecological and socioeconomic effects of ecological restoration in China's Three Rivers Source Region. Science of the Total Environment, 650, 2307–2313. 10.1016/j.scitotenv.2018.09.265 30292990

[ece35906-bib-0046] Shipley, B. (2000). A new inferential test for path models based on directed acyclic graphs. Structural Equation Modeling, 7, 206–218. 10.1207/S15328007SEM0702_4

[ece35906-bib-0047] Song, W. , Deng, X. , Yuan, Y. , Wang, Z. , & Li, Z. (2015). Impacts of land‐use change on valued ecosystem service in rapidly urbanized North China Plain. Ecological Modelling, 318, 245–253. 10.1016/j.ecolmodel.2015.01.029

[ece35906-bib-0048] Stahlberg, S. (1980). New extraction method for estimation of plant‐available P, K and Mg‐trial application in swedish cultivated soils. Acta Agriculturae Scandinavica, 30, 93–107.

[ece35906-bib-0049] Steffen, W. , Richardson, K. , Rockstrom, J. , Cornell, S. E. , Fetzer, I. , Bennett, E. M. , … Sörlin, S. (2015). Sustainability. Planetary boundaries: Guiding human development on a changing planet. Science, 347, 1259855. 25592418 10.1126/science.1259855

[ece35906-bib-0050] Sun, W. , Song, X. , Mu, X. , Gao, P. , Wang, F. , & Zhao, G. (2015). Spatiotemporal vegetation cover variations associated with climate change and ecological restoration in the Loess Plateau. Agricultural and Forest Meteorology, 209, 87–99. 10.1016/j.agrformet.2015.05.002

[ece35906-bib-0051] Temperton, V. , Mwangi, P. , Scherer‐Lorenzen, M. , Schmid, B. , & Buchmann, N. (2007). Positive interactions between nitrogen‐fixing legumes and four different neighbouring species in a biodiversity experiment. Oecologia, 151, 190–205. 10.1007/s00442-006-0576-z 17048010

[ece35906-bib-0052] Tscharntke, T. , Tylianakis, J. M. , Rand, T. A. , Didham, R. K. , Fahrig, L. , Batáry, P. , … Westphal, C. (2012). Landscape moderation of biodiversity patterns and processes ‐ Eight hypotheses. Biological Reviews of the Cambridge Philosophical Society, 87, 661–685. 10.1111/j.1469-185X.2011.00216.x 22272640

[ece35906-bib-0053] Valliere, J. M. , Zhang, J. , Sharifi, M. R. , & Rundel, P. W. (2019). Can we condition native plants to increase drought tolerance and improve restoration success? Ecological Applications, 29, e01863 10.1002/eap.1863 30831005

[ece35906-bib-0054] Walkley, A. , & Black, I. A. (1934). An examination of the Degtjareff method for determining soil organic matter, and a proposed modification of the chromic acid titration method. Soil Science, 37, 29–38. 10.1097/00010694-193401000-00003

[ece35906-bib-0055] Wang, C. , Ma, Y. , Trogisch, S. , Huang, Y. , Geng, Y. , Scherer‐Lorenzen, M. , & He, J.‐S. (2017). Soil respiration is driven by fine root biomass along a forest chronosequence in subtropical China. Journal of Plant Ecology, 10, 36–46. 10.1093/jpe/rtw044

[ece35906-bib-0056] Wang, C. , & Tang, Y. (2019). A global meta‐analyses of the response of multi‐taxa diversity to grazing intensity in grasslands. Environmental Research Letters, 14, 114003 10.1088/1748-9326/ab4932

[ece35906-bib-0057] Wang, C. , Wang, X. , Liu, D. , Wu, H. , Lü, X. , Fang, Y. , … Bai, E. (2014). Aridity threshold in controlling ecosystem nitrogen cycling in arid and semi‐arid grasslands. Natture Communications, 5, 4799 10.1038/ncomms5799 25185641

[ece35906-bib-0058] Wang, D. , Maughan, M. W. , Sun, J. , Feng, X. , Miguez, F. , Lee, D. , & Dietze, M. C. (2012). Impact of nitrogen allocation on growth and photosynthesis of Miscanthus (Miscanthus × giganteus). Global Change Biology‐Bioenergy, 4, 688–697.

[ece35906-bib-0059] Wrighton, K. C. , Agbo, P. , Warnecke, F. , Weber, K. A. , Brodie, E. L. , DeSantis, T. Z. , … Coates, J. D. (2008). A novel ecological role of the Firmicutes identified in thermophilic microbial fuel cells. The ISME Journal, 2, 1–10. 10.1038/ismej.2008.48 18769460

[ece35906-bib-0060] Yan, D. , Mills, J. G. , Gellie, N. J. C. , Bissett, A. , Lowe, A. J. , & Breed, M. F. (2018). High‐throughput eDNA monitoring of fungi to track functional recovery in ecological restoration. Biological Conservation, 217, 113–120. 10.1016/j.biocon.2017.10.035

[ece35906-bib-0061] Zhu, B. , Gutknecht, J. L. M. , Herman, D. J. , Keck, D. C. , Firestone, M. K. , & Cheng, W. (2014). Rhizosphere priming effects on soil carbon and nitrogen mineralization. Soil Biology and Biochemistry, 76, 183–192. 10.1016/j.soilbio.2014.04.033

