# Supplementary material for: Revegetation by sowing reduces soil bacterial and fungal diversity
Source: Ecol Evol. 2019 Dec 8;10(1):431–40. doi: 10.1002/ece3.5906 (PMC6972832; doi:10.1002/ece3.5906)

**Table S1 Diversity index of plant and microbes in different restoration methods.**

| Index | Natural recovery | Sown treatment |
| --- | --- | --- |
| Richness of plant (Sp.) | 9.33±0.61b | 7.47±0.44a |
| Sobs of fungi (Per 1000 OTUs) | 217±30b | 166±8.8a |
| Sobs of bacteria (Per 1000 OTUs) | 1192±17b | 1090±21a |
| Shannon-Wiener index of plant | 0.81±0.18 | 0.60±0.07 |
| Shannon-Wiener index of fungi | 3.99±0.29 | 3.61±0.11 |
| Shannon-Wiener index of bacteria | 7.01±0.03b | 6.81±0.04a |
| Plant coverage (%) | 63.3±5.7 | 70.3±2.8 |
| Plant density (num m^-2^) | 267.8±94a | 668.4±115a |
| Pielou evenness of plant | 0.41±0.11 | 0.30±0.04 |
| Relative abundance of DS (%) | 55.5±9.5a | 81.2±3.3b |

Different letters (a and b) within the same row indicate significant differences among restoration methods (one-way ANOVA). Data shown are means ± SE (*n* = 6 or 21). Sown treatment including three species sowing: MS, *Medicago sativa* L sowing; BS, *Bromus inermis Leyss* sowing; AS, *Agropyron cristatum* Gaertn sowing.

**Table S2 Relative abundance of fungi at phylum levels in different restoration methods**

| Fungi | NR | MS | BS | AS |
| --- | --- | --- | --- | --- |
| *Ascomycota* | 80.11±3.59a | 85.63±2.56b | 82.76±5.01ab | 78.99±5.16a |
| *Basidiomycota* | 8.63±1.36a | 7.19±1.26a | 8.50±2.15a | 7.28±1.41a |
| *Chytridiomycota* | 0.17±0.08a | 0.33±0.15a | 0.22±0.10a | 0.11±0.03a |
| *Glomeromycota* | 0.42±0.10b | 0.69±0.39b | 0.53±0.18b | 0.19±0.09a |
| *Zygomycota* | 7.99±2.84b | 4.37±1.17a | 6.02±3.15ab | 10.98±5.41b |

Different letters (a and b) within the same row indicate significant differences among restoration methods (one-way ANOVA). Data shown are means ± SE (*n* = 6 or 9). NR, natural recovery; MS, *Medicago sativa* L sowing; BS, *Bromus inermis Leyss* sowing; AS, *Agropyron cristatum* Gaertn sowing.

**Table S3 Relative abundance of bacteria at phylum levels in different restoration methods**

| Bacteria | NR | MS | BS | AS |
| --- | --- | --- | --- | --- |
| *Acidobacteria* | 16.45±1.48b | 13.87±1.02a | 14.14±1.25a | 16.35±1.10b |
| *Actinobacteria* | 20.41±1.29a | 21.30±0.89a | 21.82±2.21a | 22.41±1.19a |
| *Bacteroidetes* | 5.48±0.94a | 7.29±1.39a | 7.01±1.19a | 5.35±0.67a |
| *Chloroflexi* | 9.58±1.10a | 8.94±0.98a | 9.65±1.11a | 9.50±0.32a |
| *Firmicutes* | 4.92±1.15b | 1.24±0.48a | 1.87±0.28a | 0.96±0.16a |
| *Gemmatimonadetes* | 4.48±0.21a | 3.73±0.56a | 3.75±0.41a | 4.35±0.27a |
| *Proteobacteria* | 30.33±2.33a | 32.53±3.05a | 31.88±2.31a | 32.93±2.71a |

Different letters (a and b) within the same row indicate significant differences among restoration methods (one-way ANOVA). Data shown are means ± SE (*n* = 6 or 9). NR, natural recovery; MS, *Medicago sativa* L sowing; BS, *Bromus inermis Leyss* sowing; AS, *Agropyron cristatum* Gaertn sowing.

**Table S4 The results of ANCOVA for the relationship between biodiversity and environmental factors.**

| Num | Environmental factors | Plant diversity | | Fungal diversity | | | Bacterial diversity | |
| --- | --- | --- | --- | --- | --- | --- | --- | --- |
|  |  | *F* | *P* | *F* | *P* | | *F* | *P* |
| 1 | **Fungal diversity** | **12.95** | **＜0.01** |  |  | |  |  |
|  | Restoration methods | **3.08** | **0.04** |  |  | |  |  |
| 2 | **Bacterial diversity** | **8.86** | **＜0.01** | **3.65** | **0.07** | |  |  |
|  | Restoration methods | **4.06** | **0.02** | 1.13 | 0.36 | |  |  |
| 3 | **Plant density** | 9.35 | ＜0.01 |  |  | |  |  |
|  | Restoration methods | 1.99 | 0.15 |  |  | |  |  |
| 4 | **Available potassium** | **4.71** | **0.04** |  |  | |  |  |
|  | Restoration methods | **3.65** | **0.03** |  |  | |  |  |
| 5 | **Soil moisture** |  |  | **2.92** | **0.10** | |  |  |
|  | Restoration methods |  |  | 1.17 | 0.34 | |  |  |
| 6 | **Soil pH** |  |  |  | |  | **5.51** | **0.02** |
|  | Restoration methods |  |  |  | |  | 0.21 | 0.89 |
| 7 | **Available phosphorus** |  |  |  | |  | **2.99** | **0.09** |
|  | Restoration methods |  |  |  | |  | 0.42 | 0.74 |

Restoration methods as a co-variable in the model.

**Table S5 Sobs of *Zygomycota* in fungi and *Firmicutes* in bacteria among different restoration methods.**

| Phylum | NR | MS | BS | AS |
| --- | --- | --- | --- | --- |
| *Zygomycota* | 8.1±1.1c | 5.2±0.7a | 6.8±0.6bc | 6.5±0.6b |
| *Firmicutes* | 37.5±4.7c | 30.8±2.2b | 34.2±3.5bc | 26.7±1.2a |

Different letters (a, b, and c) within the same row indicate significant differences among restoration methods (one-way ANOVA). Data shown are means ± SE (*n* = 6 or 9). NR, natural recovery; MS, *Medicago sativa* L sowing; BS, *Bromus inermis Leyss* sowing; AS, *Agropyron cristatum* Gaertn sowing.

**Figure S1** Photos for different restoration treatment after one-year experiment.


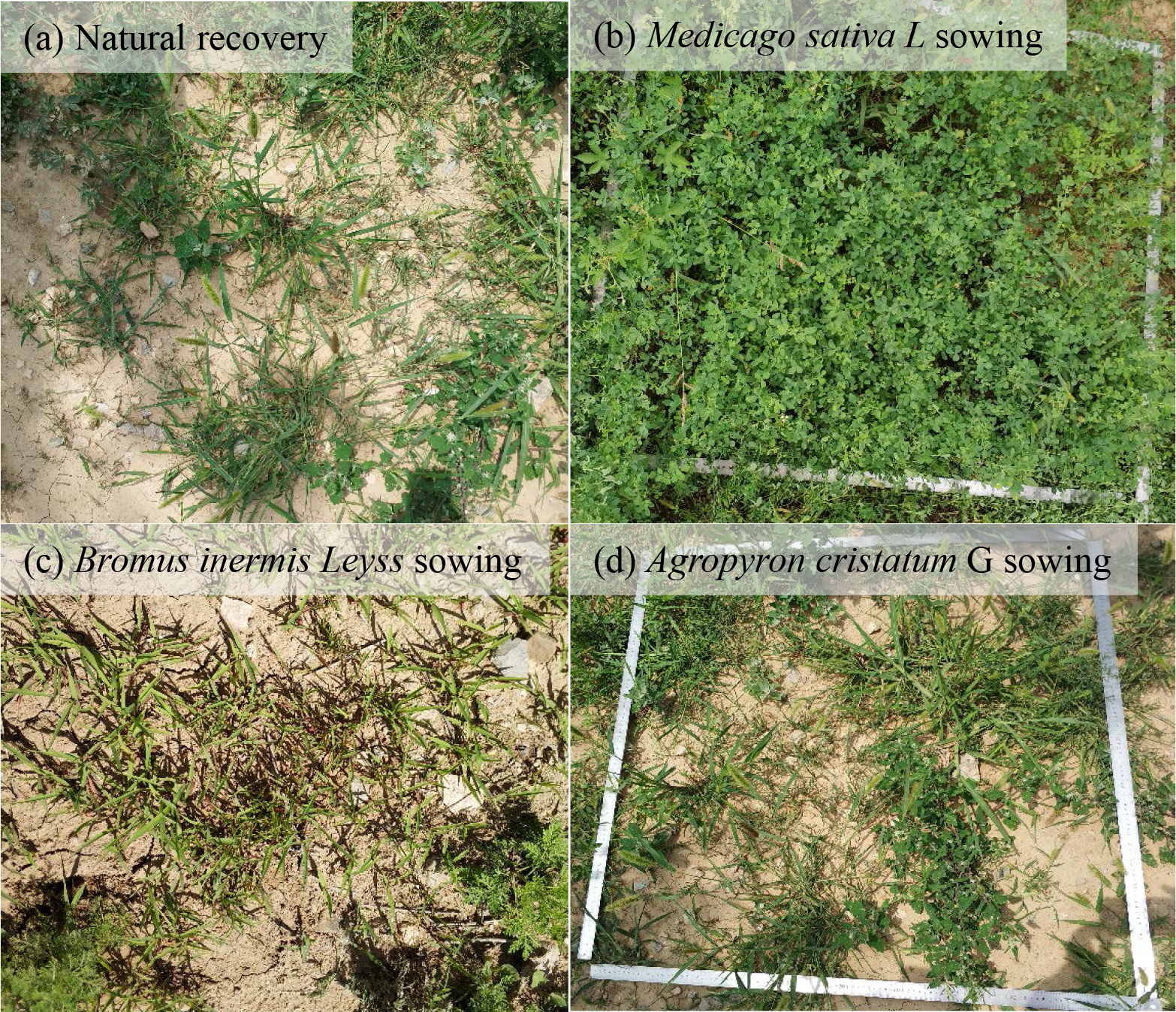

Supplement: Supplementary file 1 [file ECE3-10-431-s001.docx]
